# Anti-β2-glycoprotein I and anti-phosphatidylserine/prothrombin antibodies exert similar pro-thrombotic effects in peripheral blood monocytes and endothelial cells

**DOI:** 10.1186/s13317-019-0113-9

**Published:** 2019-04-06

**Authors:** A. Cifù, R. Domenis, C. Pistis, F. Curcio, M. Fabris

**Affiliations:** 1 Deparment of Medical Area (DAME), University of Udine, Udine, Italy; 2 Department of Laboratory Medicine, Institute of Clinical Pathology, University Hospital of Udine, Via Chiusaforte, Ingresso H, 33100 Udine, Italy

**Keywords:** Anti-phosphatidylserine/prothrombin, Anti-β2-glycoprotein I, Tissue factor, Nitric oxide, Anti-phospholipid syndrome

## Abstract

**Purpose:**

The introduction of the anti-phosphatidylserine/prothrombin (aPS/PT) antibodies among the routinely investigated anti-phospholipid (aPL) antibodies led to an improvement in anti-phospholipid syndrome (APS) laboratory diagnostic performance; however, their pathogenic mechanism is still substantially undefined. To support clinical data and future inclusion as possible new criteria antibodies, we designed a head-to-head study to directly compare the procoagulant effects sustained in vitro by aPS/PT to those sustained by anti-β2-glycoprotein I (aβ2GpI) domain 1-specific antibodies.

**Methods:**

Blood donors-derived monocytes and endothelial cells (HUVEC) were stimulated with lipopolysaccharides (LPS) alone or in combination with the IgG fractions isolated from the serum of six APS patients, positive only for aβ2GpI or for aPS/PT antibodies. As control, cells were incubated with LPS plus the IgG isolated from blood donors. Tissue factor (TF) mRNA expression was measured after four hours incubation by real-time PCR. Nitric oxide (NO) levels were measured in cells supernatant after 16 h incubation by colorimetric assay.

**Results:**

aPS/PT and aβ2GpI IgG antibodies fractions showed comparable ability to enhance LPS-induced TF mRNA expression, either in monocytes and in HUVEC. Compared to LPS alone, we found that NO levels are strongly overproduced in HUVEC treated with LPS plus aβ2GpI and aPS/PT IgG fractions.

**Conclusions:**

Our data support the significant and independent role of aPS/PT in the pathogenesis of the thrombotic events in APS patients, possibly adding new light to the therapeutic management of cases characterized by the sole presence of aPS/PT IgG antibodies.

**Electronic supplementary material:**

The online version of this article (10.1186/s13317-019-0113-9) contains supplementary material, which is available to authorized users.

## Introduction

Antiphospholipid syndrome (APS) is an autoimmune disorder characterized by vascular thrombosis (venous or arterial) and/or adverse obstetric outcomes accompanied by persistent and elevated levels of antiphospholipid (aPL) antibodies. According to the 2006 revised international classification criteria, the presence of one among anti-beta2 glycoprotein I (aβ2GPI) IgG or IgM, anti-cardiolipin (aCL) IgG or IgM and the lupus anticoagulant (LAC) is required for a definite diagnosis of APS [1]. Recently, a new class of aPL, the autoantibodies recognizing the phosphatidylserine/prothrombin complex (aPS/PT), is emerging as a possible additional member of the APS classification criteria [2], due to their elevated correlation with LAC and thrombotic events [3–5]. These antibodies are not related to those against prothrombin alone; their target is a complex of anionic phospholipid and its binding protein, such as the cardiolipin-β2GpI complex.

Numbers of recent papers underlined the important role of aPS/PT antibodies, together with aβ2GPI, aCL and LAC, improving the diagnosis and prognosis of APS. Anti-PS/PT antibody assays demonstrated high diagnostic performance for patients with APS, but can also detect some APS patients negative for criteria antibodies [6] and may serve as potential risk predictors for venous thrombosis and obstetric complications [7, 8].

The introduction of the aPS/PT antibodies among the routinely investigated aPL antibodies has led to an improvement in APS laboratory diagnostic performance, as shown in a recent observational study performed in our and other laboratories [8–10].

Of note, due to their elevated correlation with LAC activity, aPS/PT could help when immunological deficits or anticoagulant therapy avoid a correct LAC interpretation [8]. However, their pathogenic mechanism is still substantially undefined and, to date, aPS/PT antibodies are not included among the diagnostic criteria of APS and only few clinical laboratories include aPS/PT in routine analyses so far. In fact, no clear recommendations are available to guide the therapeutic approach in patients positive only for aPS/PT antibodies, despite several recent evidences depicting the remarkable rate of APS diagnoses with such isolated positivity compared to the isolated aβ2GpI positivity [5].

To further support these clinical data and future inclusion as possible new criteria antibodies, we designed a head-to-head study to directly compare the biological effects sustained in vitro by aPS/PT to those sustained by aβ2GpI domain 1 specific antibodies, developing an experimental model able to investigate the basic pathogenic mechanism underlying the pro-thrombotic effects associated to the presence of these antibodies.

## Material and methods

### Patients

We selected six female patients, mean age 51.5 ± 10.4 years. Three patients were highly positive for aCL IgG and aβ2GpI IgG (93 ± 60 AU/ml), negative for aCL and aβ2Gp IgM and negative for aPS/PT IgG/IgM. All were LAC positive and all disclosed aβ2GpI domain 1-specificity. One patient was diagnosed as secondary APS (she was affected by systemic lupus erythematosus), while the other two were classified as primary APS (persistent aPL positive, in anti-aggregating therapy).

The second group of patients were instead highly positive for aPS/PT IgG (70 ± 25 AU/ml), negative for aPS/PT IgM and negative for aCL IgG/IgM and aβ2GpI IgG/IgM. One patient was LAC positive, the other two negative. One patient was diagnosed as secondary APS (she was affected by systemic lupus erythematosus), the other two as primary APS (persistent aPL positive, in anti-aggregating therapy). As controls, we enrolled five female age-matched blood donors (BD), who tested negative for LAC, aCL IgG/IgM, aβ2GpI IgG/IgM and aPS/PT IgG/IgM antibodies.

The study was performed in accordance with the principles of good clinical and laboratory practice. All patients and controls gave their informed consent to this retrospective study according to the Declaration of Helsinki and to the Italian legislation (Authorization of the Privacy Guarantor No. 9, 12th of December 2013). All samples were identified by an anonymous code.

### aPL assays

Anti-CL IgG/IgM and aβ2GpI IgG/IgM were analysed by diagnostic chemiluminescence method (Zenit RA, Menarini; cut-off 20 AU/ml); aPS/PT IgG/IgM were analysed by commercial ELISA (Inova Diagnostics; cut-off 40 AU/ml for IgG; 30 AU/ml for IgM). Anti-β2GpI domain I antibodies were tested by chemiluminescence (Inova Diagnositcs, Bioflash; cut-off 20 AU/ml). Plasma samples were tested for the presence of LAC according to the recommended criteria from the ISTH Subcommittee on Lupus Anticoagulant-Phospholipid-dependent antibodies and optimized according to recently published standardization [11].

### Isolation of IgG

Total IgG from the sera of patients and controls were purified with Rec.Protein A-Sepharose^®^ 4B Conjugate (Life Technologies) according to the manufacturer's instruction. Briefly, Rec.Protein A-Sepharose^®^ was bound to the Fc region of IgG and then, to elute IgGs, a 0.1 M glycine buffer pH 3.0 was used. To preserve the activity of purified IgG, the pH of fractions was neutralized by addition of 1:10 vol./vol. of 1 M Tris–HCl pH 9.0. Immunoglobulins were eluted in the first two fractions.

Protein concentration of the immunopurified IgG was directly measured using the NanoDrop 1000™-Thermo Scientific™ system, reading the absorbance at 280 nm.

The purity of immunopurified IgG was verified by sodium dodecyl sulfate–polyacrylamide gel electrophoresis (SDS-PAGE) and immunofixation, using a diagnostic assay performed on the fully automated gel electrophoresis instrument InterlabG26.

The IgG fractions obtained by each of the two groups of patients and by BD were pooled to finally obtain three IgG fractions (aβ2GpI single positive, aPS/PT single positive, aPL negative) with the same concentration of IgG in order to put the same volume in each well for all the experiments.

### Isolation of monocytes

PBMCs (Peripheral Blood Mononuclear Cells) were isolated from blood donors by gradient centrifugation (Ficoll-Paque Plus, GE Healthcare) and monocytes were purified by negative selection using the Human Monocyte Enrichment Kit (Stemcell Technologies) according to the manufacturers’ suggested protocol. Isolated monocytes were cultured overnight in RPMI-1640 (Sigma-Aldrich) supplemented with 0.1 M HEPES (Sigma-Aldrich), 100 μM penicillin–streptomycin (Sigma-Aldrich), and 10% heat-inactivated Fetal Bovine Serum (FBS, Gibco) in humidified atmosphere (5 vol.% CO_2_, 37 °C).

### HUVEC culture condition

Human Umbilical Vein Endothelial Cells (HUVEC) (Gibco), were maintained under 5 vol.% CO_2_ at 37 °C in M200 (Gibco) supplemented with 100 μM penicillin–streptomycin (Sigma-Aldrich), and Low Serum Growth Supplement (LSGS; Gibco).

## Treatment with purified IgG fraction from patients and controls

To mimic the “two-hit” theory, cells were stimulated with lipopolysaccharide (LPS from *Escherichia coli* O55:B5, Sigma-Aldrich) at concentration of 1 ng/ml plus the pooled IgG fractions (final concentration 0.5 mg/ml) from patients (aβ2GpI+ or aPS/PT+) and controls (BDs) [12, 13]. Then, prothrombin (PT, BioVision) was added to the medium (10 μg/ml for monocytes and 15 μg/ml for HUVEC), since β2GpI is produced by monocytes and HUVEC under LPS-stimulation, while PT is produced only by liver cells [14, 15]. To facilitate the binding of PT to phosphatidylserine, the calcium concentration was adjusted to 2.5 mM, as suggested by Oku et al. [15]. Monocytes and HUVEC incubation lasted four hours for mRNA analysis, while HUVEC were incubated 16 h to test nitric oxide (NO) production.

### RNA isolation and real-time PCR

Total RNA was extracted from the cells using ReliaPrep™ RNA Cell Miniprep System according to the manufacturer’s protocol and stored at − 80 °C until use. The NanoDrop™ 1000 Spectrophotometer (ThermoFisher Scientific) was used to assess RNA quality and quantity.

Complementary DNA (cDNA) was generated using the iScript™ Select cDNA Synthesis Kit (Bio-Rad Laboratories) according to the random primer protocol provided by the manufacturer.

Tissue factor (TF) mRNA relative expression was analysed by real-time PCR using SsoAdvance Universal SYBR green super mix (Bio-Rad Laboratories) and LightCycler 480 (Roche Diagnostics Ltd) according to the manufacturer’s instructions. The results of mRNA expression were analysed by measuring threshold cycle and the value was normalized with GAPDH using ΔΔct method. The sequences of the primers used in this study are the following: TF forward 5′ TGTTCAAATAAGCACTAAGTCAGGAGAT 3′ and reverse 5′ TCGTCGGTGAGGTCACACTCT 3′ and GAPDH forward: 5′ AGTATGACAACAGCCTCAAG 3′, primer reverse 5′ TCTAGACGGCAGGTCAGGTCCAC 3′.

### Measurement of nitric oxide (NO) production

HUVEC were stimulated for sixteen hours and supernatants were collected, centrifuged at 15,500×*g* (5 min, 4 °C) and stored at − 80 °C until NO quantification.

The measurement of NO levels was performed using a colorimetric assay (Nitrate/Nitrite Colorimetric Assay Kit-Cayman Chemical Company, Ann Arbor, Michigan, USA) according to the manufacturers’ instruction. Considering that NO is scavenged rapidly, the final products (NO_x_) in vivo are nitrite (${\text{NO}}_{2}^{ - }$) and nitrate (${\text{NO}}_{3}^{ - }$), thus the best index of total NO production is the sum of nitrite and nitrate.

### Statistical analysis

Statistical analysis has been performed using GraphPad Software (version 5). Data were tested for normal distribution using the Kolmogorov–Smirnov test. Since they were not normally distributed, repeated measurements were analysed by one-way analysis of variance followed by the Dunnett post-test. p values less than 0.05 were considered significant. Quantitative variables were expressed as mean ± standard deviation (SD).

## Results

### IgG fraction isolation from sera

**Fig. 1:**
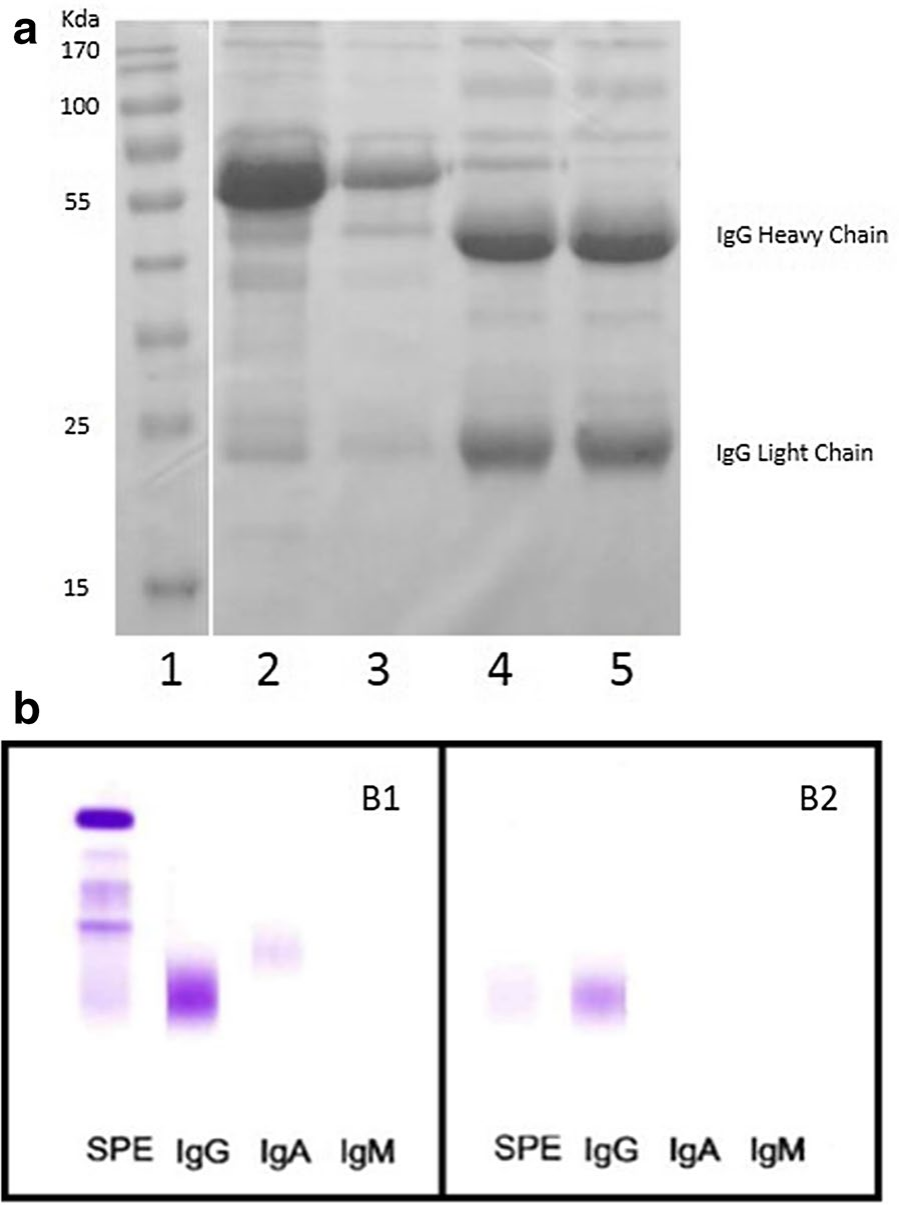
**a** Purification of IgG from human serum with affinity chromatography: lines 1 shows molecular weight markers, lane 2 shows the input material (serum), lane 3 shows serum depleted from IgG, lanes 4 and 5 show the bound IgG fraction. **b** Immunofixation of the serum, the input material (b1), and IgG fraction (b2): as shown the fraction of IgG is highly pure. *SPE* serum protein electrophoresis

The absence of contaminants of the IgG fractions isolated from patients’ and controls’ sera by affinity chromatography was checked either by SDS-PAGE and immunofixation. As shown in Fig. 1a, the presence of IgG heavy and light chain indicates that eluates were enriched in IgG. The absence of contamination by other immunoglobulins (IgA and IgM) was verified by immunofixation (Fig. 1b). To verify that the purification process did not alter the antibody specificity, we measured aβ2GpI IgG and aPS/PT IgG antibodies in the IgG fraction purified from patient sera, finally confirming the presence of the respective antibodies at high titre, while the IgG fraction isolated from BD sera remained negative (data not shown).

## TF monocyte expression modulation by purified IgG fractions of patients and controls

**Fig. 2:**
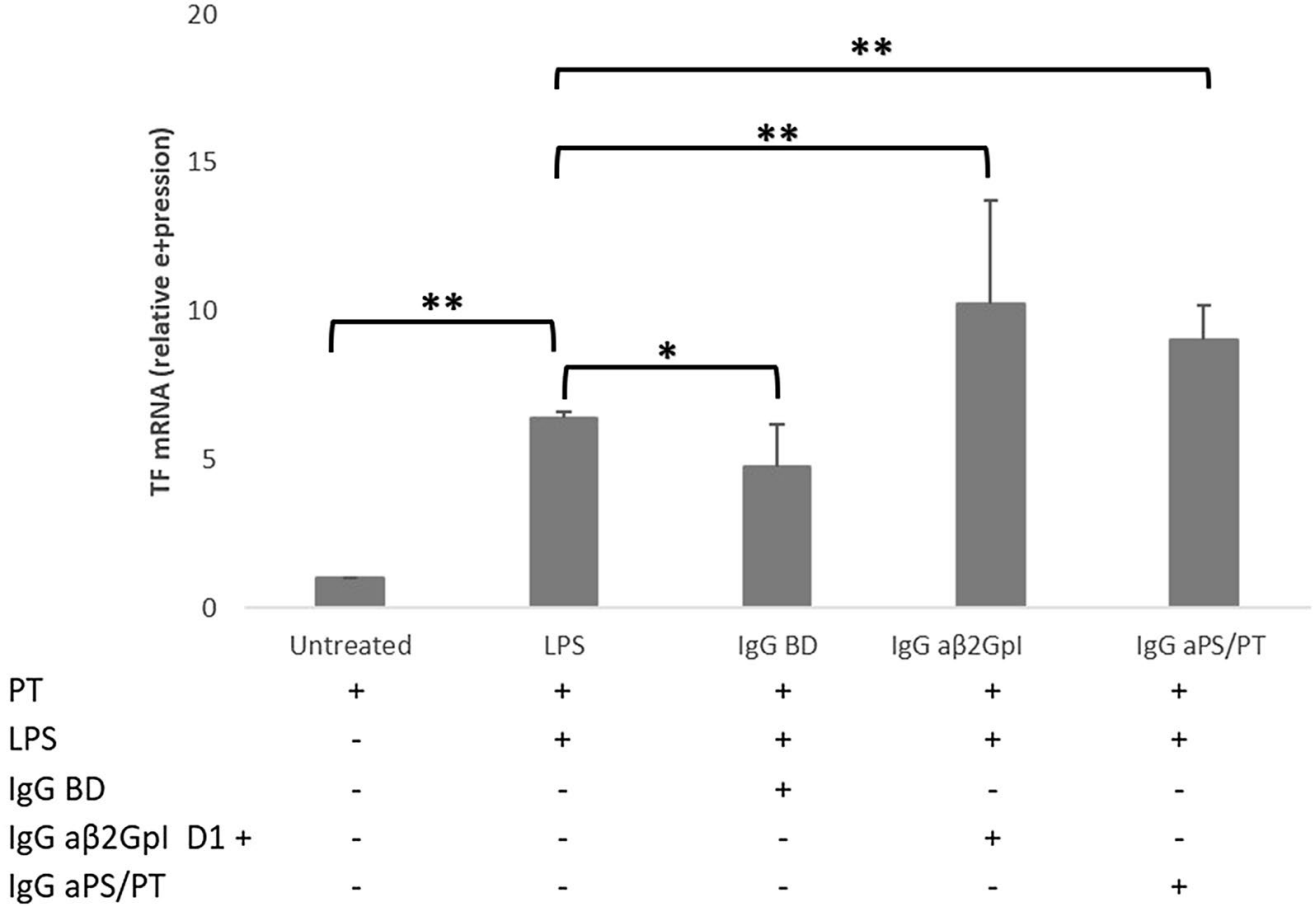
TF mRNA relative expression levels in monocytes obtained from PBMCs of five BD stimulated as described below for 4 h. PT was added in all cases, while LPS (1 ng/ml) was added alone or followed by the IgG fractions (0.5 mg/ml) extracted from three BD or aPL + patients disclosing aβ2GpI positive antibodies (three pooled sera) or aPS/PT positive antibodies (three pooled sera). The bars represent the mean ± SD of three independent experiments. The expression levels of mRNA were detected by PCR real-time with ΔΔct method. *p < 0.05, **p < 0.001

As shown in Fig. 2, compared to the untreated cells, TF mRNA expression was found to be significantly increased by treatment with LPS (6.4 ± 0.2 relative expression). Of note, treatment with LPS plus the IgG fractions isolated from BD increased TF mRNA expression significantly less than LPS alone (4.7 ± 1.4 vs 6.4 ± 0.2; p = 0.015). In contrast, treatment with LPS plus the IgG fractions isolated from APS patients, either expressing aβ2GpI, or aPS/PT antibodies, significantly up-regulated TF mRNA expression compared to LPS alone (10.2 ± 3.5 for aβ2GpI, p = 0.007 and 9 ± 1.1 for aPS/PT, p = 0.00001) and no significant difference was found between aβ2GpI and aPS/PT antibodies.

### TF HUVEC expression modulation by purified IgG fractions isolated from patients and controls

**Fig. 3:**
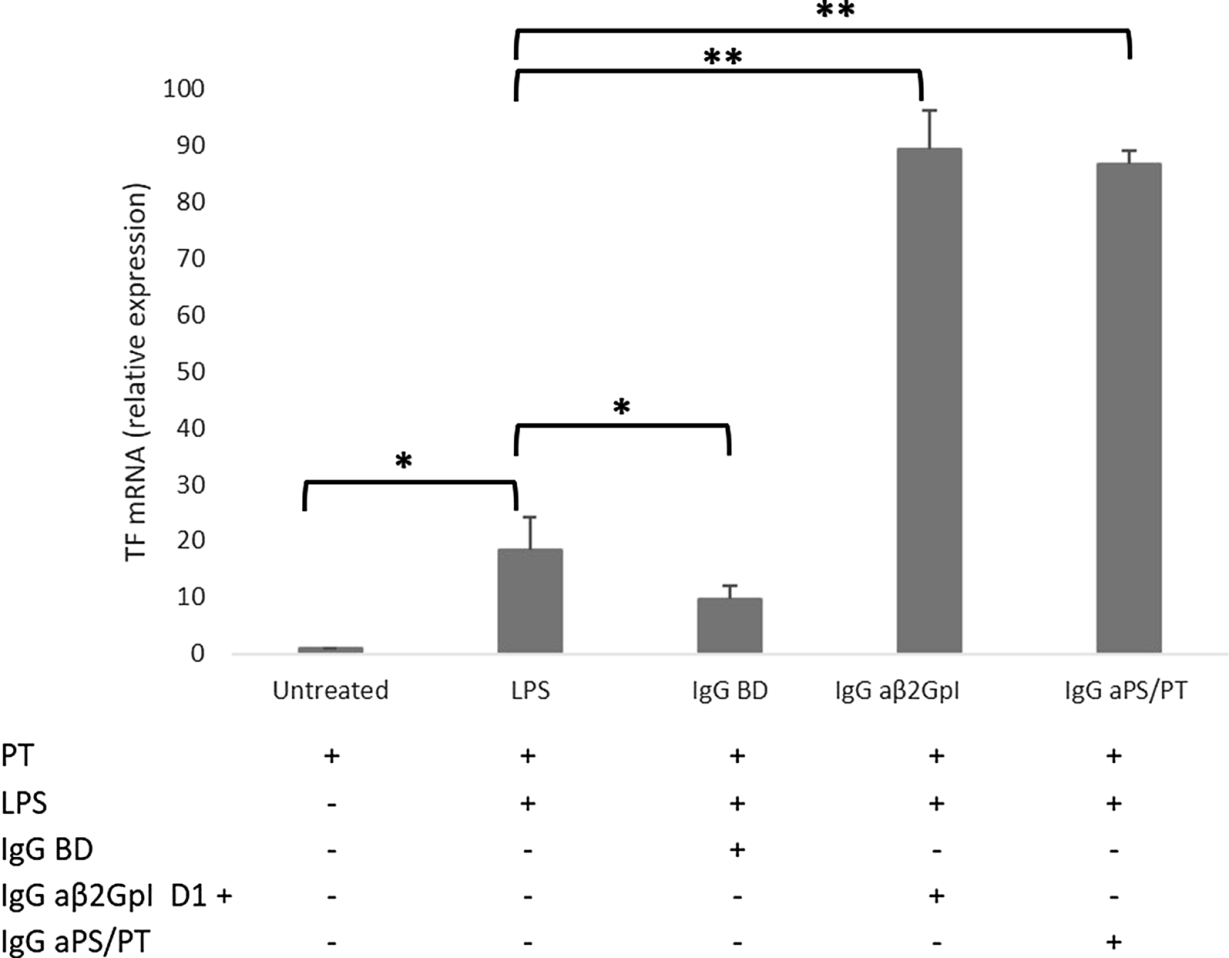
TF mRNA relative expression in HUVEC. Cells were stimulated for four hours with LPS (1 ng/ml) alone or with the IgG fractions (0.5 mg/ml) extracted from three BD or aPL + patients disclosing aβ2GpI positive antibodies (three pooled sera) or aPS/PT positive antibodies (three pooled sera). The bars represent the mean ± SD of three independent experiments. The expression levels of mRNA were detected by PCR real-time with ΔΔct method. *p < 0.05, **p < 0.001

As for monocytes, HUVEC were treated with LPS alone or in combination with the IgG fractions isolated from sera of BD or APS patients positive for aβ2GpI or aPS/PT IgG antibodies (Fig. 3). Treatment of HUVEC with LPS plus BD IgG fraction determined a downregulation of TF mRNA expression compared to the LPS stimulation alone (9.6 ± 2.4 vs 18.4 ± 5.7; p = 0.004). In contrast, the incubation with LPS plus the IgG fractions from APS patients caused a significant increase (89.5 ± 6.7, p = 0.00001 for aβ2GpI and 86.8 ± 2.4, p = 0.00001 for aPS/PT, respectively) of TF mRNA expression, and no significant difference was observed between aβ2GpI+ and aPS/PT+ antibodies (Fig. 3).

### Nitric oxide production by HUVEC after IgG fraction stimulation

**Fig. 4:**
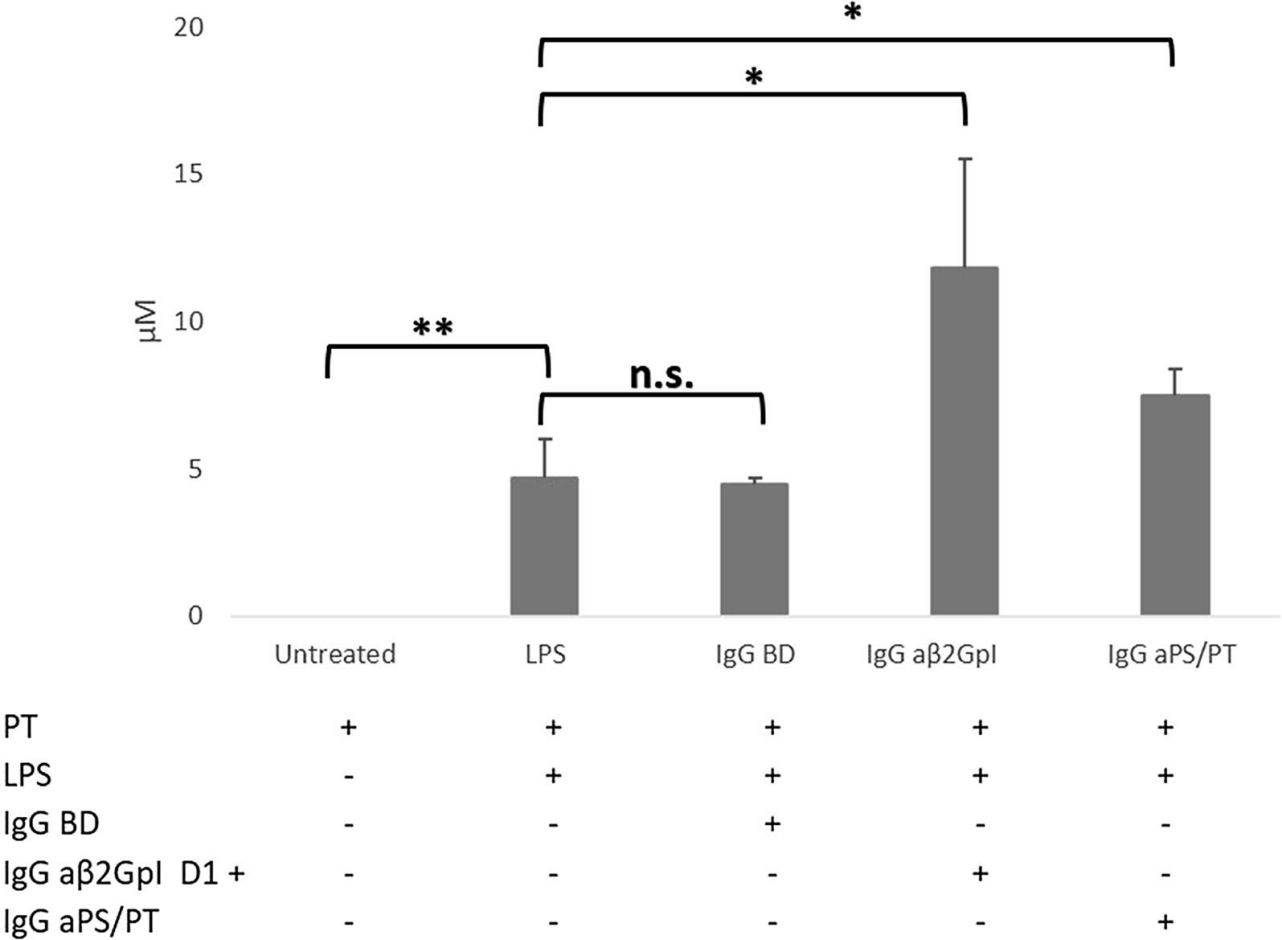
NO production in HUVEC. Cells were stimulated for sixteen hours with LPS (1 ng/ml) alone or with the IgG fractions (0.5 mg/ml) extracted from three BD or aPL + patients disclosing aβ2GpI positive antibodies (three pooled sera) or aPS/PT positive antibodies (three pooled sera). The bars represent the mean ± SD of three independent experiments. NO_x_ levels were measured in supernatant of untreated and treated-cells. *p < 0.05, **p < 0.001

The treatment with LPS induced NO_x_ production by endothelial cells (Fig. 4), but no difference was found between treatment with LPS alone or plus BD IgG fraction (4.7 ± 1.3 µM vs 4.5 ± 0.18 µM; p = 0.86). In contrast, NO_x_ levels were significantly up-regulated by treatment with LPS plus aβ2GpI+ and aPS/PT + IgG fractions (respectively 11.8 ± 3.7 µM for aβ2GpI+ , p = 0.03 and 7.5 ± 0.9 µM for aPS/PT, p = 0.035). NO_x_ release tended to be higher with aβ2GpI than with aPS/PT, but the difference was not significant.

## Discussion

Anti-PS/PT antibodies are frequently cited as the most promising candidate for the upcoming revision of the APS diagnostic criteria [2]. They were often compared with the aβ2GpI antibodies when analysing prognostic role, clinical correlation, persistence, LAC association, with overall performance generally less impressive than the classical triple positivity.

Several studies already documented the procoagulant effect of anti-β2GpI by analysing TF expression upregulation in monocytes and endothelial cells [16–18]. Recently, Benagiano et al., have shown the presence of plaque-derived CD4^+^ cell clones specific for β2GpI, suggesting that anti-β2GpI antibodies not only have a role in the acquired procoagulant diathesis, but also in the accelerated atherosclerosis typically found in patients with primary and secondary APS, as well as in lupus patients [19].

Much less is actually known regarding anti-PS/PT antibodies, apart from the elegant study of Oku et al. who showed that the expression of TF in procoagulant cells was induced by aPS/PT via p38MAPK phosphorylation, using both the IgG fractions from APS patients and a murine monoclonal aPS/PT antibody [15].

At our knowledge, no direct comparison was performed between aβ2GpI and aPS/PT antibodies.

In our study, for the first time, we tested under the same experimental conditions the procoagulant effect in vitro of anti-PS/PT antibodies versus the anti-β2GpI antibodies, finding comparable ability to enhance TF expression, both in monocytes and in endothelial cells. Both aPS/PT and aβ2GpI IgG antibodies were isolated from patients diagnosed as APS. Of note, all the aβ2GpI IgG positive sera disclosed high positive reactivity against the domain 1 of the β2GpI. Thus, after a classical inflammatory activation (LPS), isolated aPS/PT antibodies are able to induce TF expression at a very similar extent compared to aβ2GpI, both in monocytes and in HUVEC. Both resulted stronger than LPS alone. We also replicated the experiment in monocytes using single sera from two selected patients with primary APS (PAPS), one showing high title of aβ2GpI IgG (155 AU/ml) with Domain 1-specificity, the other high positive aPS/PT IgG (150 AU/ml), LAC positive and negative aCL and aβ2GpI IgG/IgM. The results confirmed data with pooled sera (see Additional file 1).

Our results partially confirmed those presented by Oku et al. [15], since they incubated PBMCs or HUVEC directly with aPS/PT isolated from APS patients or a murine monoclonal aPS/PT antibody, without any pre-treatment with LPS. Compared to the BD-IgG treated cells, the relative expression of TF mRNA was very low (around 4–5 times) and appeared much less than that induced by bursting with LPS alone. In our hands, treatment with aPS/PT or aβ2GpI antibodies isolated from patient samples were unable to induce any TF expression up-regulation in vitro in PBMCs or HUVEC (data not shown), unless pre-treated with low doses of LPS. This appeared in line with the accredited “two-hit” hypothesis for APS pathogenesis, that postulates the persistence of elevated levels of aPL antibodies is a necessary condition, but the occurrence of APS is seemingly triggered by an additional “second hit”, such as trauma or infection [20, 21].

Furthermore, to confirm the effect on HUVEC, we analysed the release of a specific endothelial activation factor, such as NO. Previous studies proved that LPS stimulation induced NO production in HUVEC [22], but, compared to LPS alone, we have found that NO levels are strongly overproduced in HUVEC treated with LPS plus aβ2GpI and aPS/PT IgG fractions. In this experiment, aβ2GpI antibodies tended to be inducers stronger than aPS/PT, but the difference was not significant.

To test the specificity of the effect of aPL, in our experiments we always introduced the IgG fraction isolated from a pool of blood donors. Of note, either in monocytes, but more significantly in HUVEC, the IgG fraction from BDs partially contrasted TF mRNA induction mediated by LPS alone, thus resulting even more significantly different from the effect exerted by LPS plus aPS/PT and aβ2GpI antibodies.

Probably this protective effect is due to the capability of polyclonal unspecific IgGs to buffer the pro-inflammatory molecules induced by LPS in a physiological state, as IgG isolated from BDs will represent in this experimental contest.

The relevance of the sole aPS/PT positivity for APS diagnosis is still a matter of debate. However, literature data appear increasingly in favour. As reported by Shi et al. [4], there are a significant percentage (around 27%) of the so-called seronegative APS, finally resulting positive only for anti-PS/PT IgG antibodies. Hoxha et al. [23] found aPS/PT in 9.4% of patients with clinical features suggestive of APS but negative for aPL criteria tests. In the large study of Amengual et al. [3], all APS patients from the initial cohort have APS laboratory criteria, precluding evaluation of the potential ‘added value’ of aPS/PT testing to the diagnosis of APS. But, in the replication cohort, in patients with clinical APS without positive aPL criteria tests, IgG aPS/PT were detected in one patient (6%) with thrombosis and pregnancy complications. Generally, higher titres of IgG aPS/PT were found in people with suspicion of or definite APS than in the other groups of individuals. Thus, many Authors finally conclude that in case of APS suspicion and absence of criterial aPL, testing for aPS/PT might contribute to support the APS diagnosis.

In conclusion, our data lend support to the important and independent role of aPS/PT in the pathogenesis of the thrombotic events in APS patients, possibly adding new light for the therapeutic management of cases characterized by the sole presence of aPS/PT IgG antibodies.

## Additional files

**Additional file 1: Figure S1.** TF mRNA expression in monocytes stimulated with aPS/PT IgG or aβ2GpI IgG isolated from two PAPS patients.
