# Supplementary figures and images for: Anti-β2-glycoprotein I and anti-phosphatidylserine/prothrombin antibodies exert similar pro-thrombotic effects in peripheral blood monocytes and endothelial cells
Source: Auto Immun Highlights. 2019 Apr 6;10(1):3. doi: 10.1186/s13317-019-0113-9 (PMC6909027; doi:10.1186/s13317-019-0113-9)

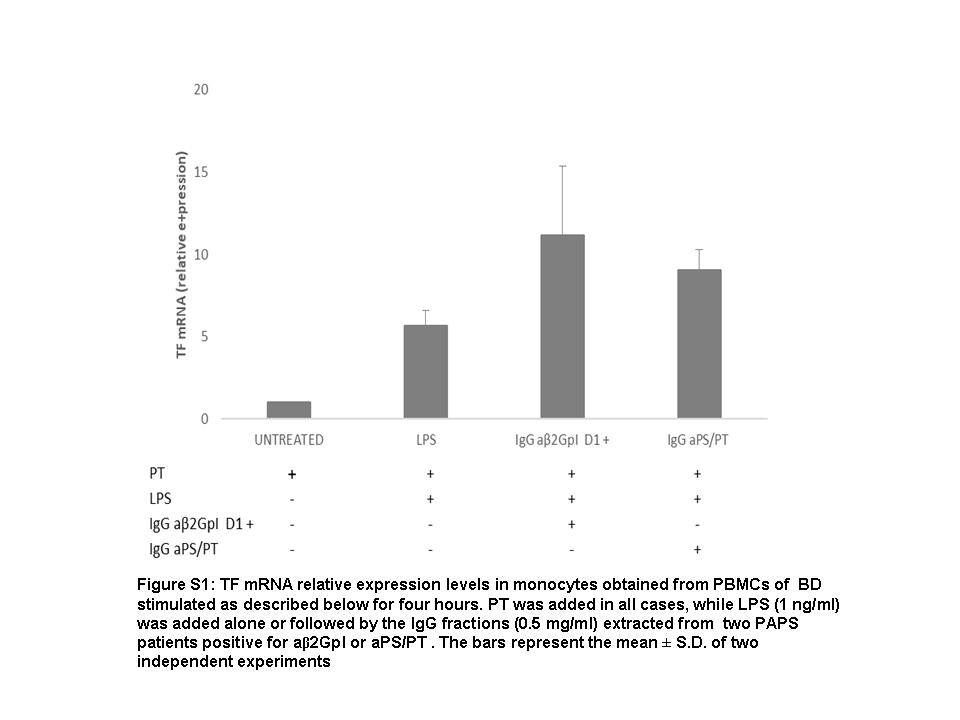

Supplement: Supplementary file 1 — Additional file 1: Figure S1. TF mRNA expression in monocytes stimulated with aPS/PT IgG or aβ2GpI IgG isolated from two PAPS patients. [file 13317_2019_113_MOESM1_ESM.jpg]
